# Label-free, real-time detection of perineural invasion and cancer margins in a murine model of head and neck cancer surgery

**DOI:** 10.1038/s41598-022-16975-w

**Published:** 2022-07-27

**Authors:** Kenric Tam, Yazeed Alhiyari, Shan Huang, Albert Han, Oscar Stafsudd, Ramesh Shori, Maie St. John

**Affiliations:** 1 Department of Head and Neck Surgery, David Geffen School of Medicine, University of California, Los Angeles, 10833 Le Conte Ave. 62-132 CHS, Los Angeles, CA 90095-1624 USA; 2 Department of Electrical and Computer Engineering, University of California, Los Angeles, Los Angeles, USA

**Keywords:** Head and neck cancer, Cancer imaging

## Abstract

Surgical management of head and neck cancer requires a careful balance between complete resection of malignancy and preservation of function. Surgeons must also determine whether to resect important cranial nerves that harbor perineural invasion (PNI), as sacrificing nerves can result in significant morbidity including facial paralysis. Our group has previously reported that Dynamic Optical Contrast Imaging (DOCI), a novel non-invasive imaging system, can determine margins between malignant and healthy tissues. Herein, we use an in vivo murine model to demonstrate that DOCI can accurately identify cancer margins and perineural invasion, concordant with companion histology. Eight C3H/HeJ male mice were injected subcutaneously into the bilateral flanks with SCCVIISF, a murine head and neck cancer cell line. DOCI imaging was performed prior to resection to determine margins. Both tumor and margins were sent for histologic sectioning. After validating that DOCI can delineate HNSCC margins, we investigated whether DOCI can identify PNI. In six C3H/HeJ male mice, the left sciatic nerve was injected with PBS and the right with SCCVIISF. After DOCI imaging, the sciatic nerves were harvested for histologic analysis. All DOCI images were acquired intraoperatively and in real-time (10 s per channel), with an operatively relevant wide field of view. DOCI values distinguishing cancer from adjacent healthy tissue types were statistically significant (P < 0.05). DOCI imaging was also able to detect perineural invasion with 100% accuracy compared to control (P < 0.05). DOCI allows for intraoperative, real-time visualization of malignant and healthy tissue margins and perineural invasion to help guide tumor resection.

## Introduction

Each year, approximately 54,000 patients in the United States and 650,000 patients worldwide are diagnosed with head and neck cancer^1,2^. Over 90% of head and neck cancers are squamous cell carcinoma (HNSCC) involving the mucosal surfaces of the aerodigestive tract. Several factors can diminish the head and neck cancer patient’s overall prognosis, including positive surgical margins and the presence of perineural invasion.

Successful surgical management of HNSCC involves complete resection of the tumor with negative margins. Positive surgical margins have been found to increase the rates of recurrence and patient mortality by 90%^3^. However, the need for negative margins should be carefully balanced against excessive resection of healthy tissue which can result in significant patient morbidity including speech and swallowing dysfunction. Margins are currently determined intraoperatively through a combination of surgeon experience, visual cues, palpation, and frozen section. Several intraoperative imaging techniques for margin detection are currently under investigation but often require the use of a contrast agent such as indocyanine green (ICG) to distinguish tumor from healthy surrounding tissue^4,5^. ICG has variable specificity for malignant tissue and intravenous administration of contrast agents, while for the most part well-tolerated, can result in adverse effects such as anaphylaxis^6^.

Intraoperatively, surgeons must also determine whether important cranial nerves harbor perineural invasion (PNI) by HNSCC. PNI is diagnosed in approximately 50% of mucosal HNSCC and is associated with increased local recurrence and decreased survival^7,8^. Sacrificing a nerve during cancer resection can also result in significant morbidity such as complete facial paralysis, dysarthria, dysphonia, or shoulder weakness. Intraoperative detection of PNI is currently determined by the surgeon’s visualization of gross neural invasion. In addition, frozen section histologic analysis can be performed to determine PNI intraoperatively, but this requires sectioning the nerve and thereby renders it nonfunctional. Current fluorescence imaging techniques under investigation for intraoperative nerve *detection* require the administration of contrast agents to distinguish healthy nerves from surrounding tissue^9^. To our knowledge, there are no intraoperative imaging techniques published that investigate the detection of PNI.

Our group has developed and previously described Dynamic Optical Contrast Imaging (DOCI), a novel imaging modality that acquires temporally-dependent measurements of tissue autofluorescence^10^. We have previously demonstrated that Dynamic Optical Contrast Imaging (DOCI) can distinguish HNSCC from adjacent healthy tissue with a high degree of accuracy^11^. DOCI images are captured in real-time, do not require administration of contrast agents, and offer an operatively wide field of view.

In this study, we demonstrate that DOCI can be utilized in an in vivo head and neck cancer mouse model to identify malignant tissue and guide tumor resection and margins. We further demonstrate that DOCI can accurately identify microscopic perineural invasion with a 100% sensitivity and specificity. This proof-of-concept study demonstrates that DOCI has the potential to revolutionize cancer care by allowing the surgeon to precisely determine cancer margins and the presence of perineural invasion intraoperatively. DOCI can then serve as a platform for multi-center trials and for the detection of other solid cancer margins throughout the body.

## Materials and methods

### Dynamic optical contrast imaging instrumentation

The instrumentation and setup for the DOCI system have been previously published^12^. A 365 nm ultraviolet-A light-emitting diode (UV-A LED) acts as the excitation light source. A 405 nm long-pass filter was utilized to block the excitation UV LED. Fluorescence emission is resolved into 9 band-pass filters (BPFs/channels): 405 nm, 415 nm, 434 nm, 465 nm, 494 nm, 520 nm, 542 nm, 572 nm, and 605 nm.

### DOCI image processing and analysis

DOCI image processing and calculation has been previously published by our group^10^. Briefly, a DOCI image is calculated for 9 filter channels, and an intensity image is computed for 9 BPF channels. A DOCI image is calculated by normalizing the aggregate fluorescence decay intensity by the aggregate stead-state fluorescence intensity. Data analysis was performed using MATLAB R2021b and Microsoft Office Excel. For each tissue sample, a region of interest (ROI) corresponding to a tissue type identified on frozen histology was selected. The average DOCI value for each ROI was then calculated. DOCI values for each tissue type were normalized against the tumor DOCI value. After confirming a normal distribution, a one-sample t-test was utilized for statistical analysis between different tissue types and cancer.

### In vivo murine model

The use of mice for this study was approved by the Institutional Animal Care and Use Committee (IACUC) at the University of California, Los Angeles. All methods were carried out in accordance with relevant guidelines and regulations, and all methods are reported in accordance with ARRIVE (Animal Research: Reporting In Vivo Experiments) guidelines. Eight C3H/HeJ male mice underwent subcutaneous, bilateral flank injection with 500,000 SCC7 cells (RRID:CVCL_V412), a murine head and neck cancer cell line. Tumors were allowed to grow for a period of three weeks. Fifteen tumors successfully grafted to the host. The mice were then anesthetized with weight-based dosing of ketamine as well as carprofen. Under anesthesia, the mice were sterilely prepped for a surgery and a midline incision was made through the skin; bilateral skin flaps were raised until the tumor was exposed. At this point, the mice underwent DOCI imaging to determine the extent of resection necessary. After assessing DOCI imaging, tumor resection was performed, followed by additional DOCI imaging of the tumor bed. Any residual tumor detected on DOCI was then resected. Both tumor and adjacent tissue margins were sent to the translational pathology core laboratory (TPCL) for permanent sectioning.

### Perineural invasion model

Methods for the creation of a nerve model of perineural invasion were adapted from Deborde et al.^13^. After induction of general anesthesia with weight-based ketamine, the bilateral sciatic nerves of six C3H/HeJ mice were dissected and exposed. Under a dissecting microscope and using a 33-gauge needle (Hamilton Company, Reno, NV), 3 uL of phosphate buffered saline (PBS) was slowly injected into the perineurium of the left sciatic nerve. Then, 50,000 SCC7 cells (RRID:CVCL_V412) in 3uL of PBS were injected into the perineurium of the right sciatic nerve. The skin was then re-approximated with 5-0 nylon sutures, and the mice were allowed to recover. All mice were noted to have normal limb function post-operatively.

### Sciatic nerve harvesting

On post-operative days 7, 10, and 14, the mice were again induced under general anesthesia with ketamine. The prior incisions were opened and the bilateral sciatic nerves were exposed. White light images were taken using a camera attached to the dissecting microscope. The bilateral nerves were then imaged using the DOCI technique. After imaging, the mice were euthanized with ketamine. The sciatic nerves were harvested proximally at the level of the spinal cord and distally at the end of the femur and then sent to TPCL for permanent sectioning.

## Results

### DOCI delineates margins between HNSCC and adjacent healthy tissue

**Figure 1:**
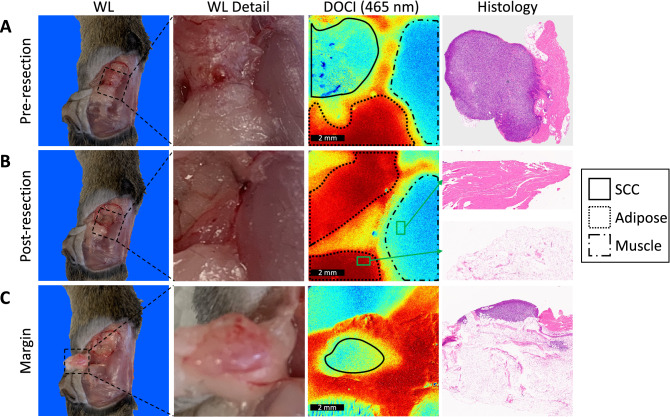
DOCI delineates margins between HNSCC and adjacent healthy tissue. (**A**) Prior to surgical resection of the tumor, DOCI was utilized to identify the tumor and determine margins. White light (WL) detail images and DOCI images were captured at a 1:1 ratio for direct comparison. Histology demonstrated HNSCC, concordant with DOCI imaging. (**B**) After resection, the tumor bed was imaged to identify any areas of tumor remaining. Histology taken from surrounding margins confirmed healthy adipose and muscle tissue (green inset). (**C**) DOCI imaging of margins revealed un-resected tumor along an adipose fat pad. Histology confirmed HNSCC with adjacent adipose fat. White light (WL), Dynamic Optical Contrast Imaging (DOCI), squamous cell carcinoma (SCC).

After surgically exposing the flank, DOCI imaging was utilized to both identify the location of the tumor and guide resection (Fig. 1). Tumor sizes ranged from 1 to 15 mm with an average size of 7 mm. Tumors were often difficult to distinguish from surrounding healthy tissue with white light (WL) alone but were clearly identified with DOCI imaging (Fig. 1A). DOCI imaging delineated the margins between HNSCC (green), compared to muscle (blue) and adipose tissue (red). Permanent histology of the tumor and adjacent healthy tissue was concordant with DOCI imaging (Figs. 1A,B). Following resection, the margins surrounding the tumor bed were re-imaged, revealing a 3 mm foci of remaining tumor inferior to the tumor bed (Fig. 1C). DOCI was able to direct further resection in this area, where the tumor was not appreciated by the surgeon’s visualization or palpation.

**Figure 2:**
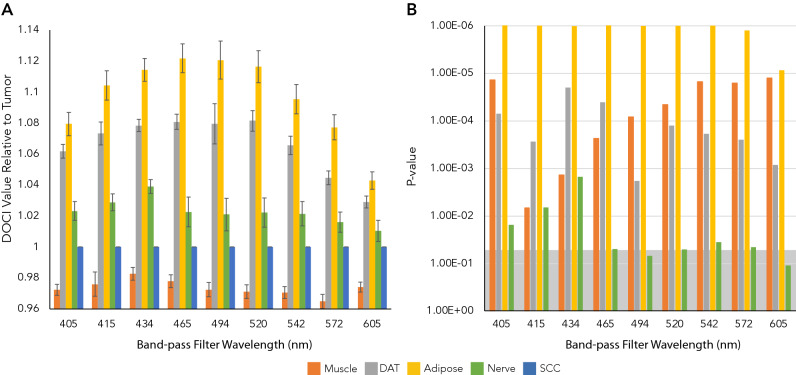
Relative lifetimes of healthy and malignant tissue. (**A**) Average relative lifetimes (DOCI values) of different tissue types as a function of wavelength (n = 15). (**B**) Manhattan plot of P values for DOCI values of each tissue type compared to HNSCC across all wavelengths. Significance is defined as P < 0.05. The significance threshold is denoted in gray and all bars above the threshold are considered significant.

The average relative lifetimes of different tissue types as a function of wavelength are shown in Fig. 2A. Each tissue type—including muscle, dermal adipose tissue (DAT), adipose tissue, nerve—has a unique DOCI value and trend relative to HNSCC. Overall, the 465 nm BPF shows the greatest contrast between tissues (Fig. 2A). *P* values were calculated for the DOCI values of each tissue type compared to SCC across all BPFs (Fig. 2B).

### DOCI accurately identifies perineural invasion

**Figure 3:**
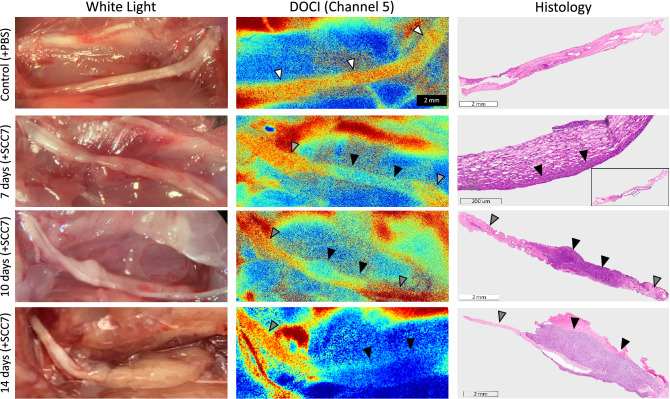
DOCI accurately identifies perineural invasion. The left sciatic nerves were injected with PBS while the right sciatic nerves were injected with SCC7. Sciatic nerves were harvested at 7, 10, and 14 days. All nerves underwent white light imaging, DOCI imaging, and permanent histologic sectioning. White arrowheads denote the control nerve, black arrowheads PNI, and grey arrowheads the normal nerve adjacent to the tumor.

After validating that DOCI can delineate HNSCC margins in our in vivo murine model, we investigated the capacity of DOCI to identify PNI. DOCI images from sciatic nerves injected with PBS were compared to sciatic nerves injected with SCC7 (Fig. 3). Control nerves had higher relative lifetimes (white arrowheads) that were consistent along the entire length of the nerve. In contrast, portions of the nerve with perineural invasion had lower relative lifetimes (black arrowheads) compared to both the control nerve as well as healthy nerve adjacent to the tumor (grey arrowheads). Histology is concordant with DOCI imaging and also demonstrates the transition point between normal nerve and PNI. Control nerves had an average diameter of 0.81 mm. PNI+ nerves had a diameter ranging from 0.91 to 2.36 mm; there was a positive correlation between post-operative day and diameter of the PNI+ portion of the nerve. Mouse hindlimb function was intact in all mice after injection.

**Figure 4:**
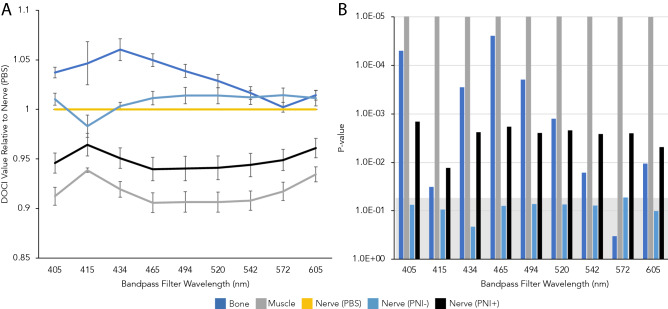
Relative lifetimes of PNI compared to healthy tissue. (**A**) Average relative lifetimes (DOCI values) of different tissue types as a function of wavelength (n = 6). PNI− nerve refers to normal nerve adjacent to tumor. (**B**) Manhattan plot of P values for DOCI values of each tissue type compared to PNI+ nerve across all wavelengths. Significance is defined as P < 0.05. The significance threshold is denoted in gray and all bars above the threshold are considered significant.

The average relative lifetimes of several regions of interest including control and PNI+ nerve are shown in Fig. 4A. Healthy nerve adjacent to tumor (PNI−) is plotted separately from the control nerve (PBS) but shows no statistically significant difference across all wavelengths (Supplementary Table 1). The P values of the different tissue types compared to PNI+ nerve are displayed in Fig. 4B as a Manhattan plot. In all BPFs, there was a statistically significant difference in DOCI values between the control and PNI+ nerves (Fig. 4B).

## Discussion

In this study, we demonstrate that DOCI can be reliably used in real-time in vivo to guide cancer resection and margins during surgery. Furthermore, DOCI can accurately identify perineural invasion, allowing the surgeon to identify in real-time which nerves need to be sacrificed and which reconstructive options are available once the affected nerve portion is removed. This is a very useful capacity as currently PNI is determined by the surgeon’s visualization of gross neural invasion. Frozen section analysis can also be utilized to detect PNI, but requires severing the nerve and thereby rendering it nonfunctional. DOCI is label-free and does not require the administration of any contrast agents. Images are generated in less than 10 s per BPF with a surgically relevant, macroscopic field of view. Lastly, DOCI images are intuitive and easy to interpret, providing the surgeon with a real time intraoperative color-coded image that delineates HNSCC from healthy tissue including nerve, adipose tissue, bone, and muscle (Figs. 1, 3).

To our knowledge, this is the first study to investigate the use of fluorescence lifetime imaging to detect perineural invasion. Prior studies in surgical fluorescent imaging have focused primarily upon the preservation of nerves and utilize contrast agents with varying nerve specificity^9^. For instance, a number of nerve-specific contrast agents have been developed, such as 4,4′-[(2-methoxy-1,4-phenylene)de-(1E)-2,1-ethenediyl]bis-benzenamine (BMB) and 4-[(1E)-2-[4-[(1E)-2-[4-aminophenyl]ethenyl]-3-methoxyphenyl]ethenyl]-benzonitrile (GE3082) which have been shown to help identify the sciatic nerve in porcine models as well as brachial plexus, sciatic, and trigeminal ganglia in rats^14,15,16^. The DOCI system does not require the use of contrast media as it is based upon the detection of fluorescence decay rates of endogenous fluorophores and thus avoids any potential toxicities that may be associated with labeling agents.

In terms of detecting PNI, the 465 nm wavelength appears to provide the greatest DOCI contrast between healthy neurons, bone, muscle, and PNI (Fig. 4A). The 465 nm wavelength also appears to provide excellent contrast between healthy tissues and HNSCC (Fig. 2A). Research into the mechanism by which DOCI detects PNI is ongoing and is likely due to alterations in the biochemical properties of HNSCC as well as a disruption of the endogenous fluorophores that are unique to healthy neurons. Malignant tissues have a number of biochemical and structural changes that occur including alteration to the concentrations of the reduced form of nicotinamide adenine dinucleotide (NADH) and flavin adenine dinucleotide (FAD). For HNSCC specifically, NADH has been determined to be the main contributor to changes in fluorescence lifetime compared to healthy tissue^17–19^.

In this proof-of-concept study, we demonstrate that DOCI can accurately detect perineural invasion of the sciatic nerve by murine SCC-7. Because DOCI detects differences in the reduced form of NADH and FAD between healthy and malignant tissues, we anticipate that PNI by other cancer cell lines will yield similar results. Furthermore, as the sciatic nerve contains both muscle and sensory nerve fibers, we predict that DOCI can likely identify PNI in sensory, motor, and mixed nerves. Trials to determine the generalizability of these results to other cell lines and nerves is ongoing.

Our group is continuously seeking to improve and refine the DOCI system. Although our study did not specifically address resolution—the smallest number of malignant cells that DOCI can detect—our imaging technique was able to distinguish normal sciatic nerves with an average of 0.81 mm and PNI+ nerves as small as 0.91 mm. Our group is actively studying the minimum threshold for DOCI and is developing new optical modalities to improve the resolution. Other future directions include the development a video DOCI system that allows for a “live view” of the operative field.

This in vivo study also demonstrates that DOCI can be utilized to guide cancer resection in real-time. DOCI was used to resect all tumors completely and there were no positive margins on permanent histology. DOCI allows for label-free, real-time visualization of malignant tissue margins and perineural invasion to help guide tumor resection. By allowing the surgeon to precisely determine margins intraoperatively, DOCI has the potential to improve patient outcomes. These results can be used as a platform for multi-center trials and for the detection of other solid cancer margins throughout the body.

## Supplementary Information

Supplementary Table 1.

## Data Availability

The datasets generated during and/or analyzed during the current study are not publicly available due to the necessity to maintain Health Insurance Portability and Accountability Act (HIPAA) compliance. The data may have patient identifiers and is not anonymized. As the data was acquired under a protocol approved by the Institutional Review Board of the University of California, Los Angeles, the IRB will need to approve data transfer to other institutions. The data are available from the corresponding author on reasonable request.
