# Supplementary material for: Label-free, real-time detection of perineural invasion and cancer margins in a murine model of head and neck cancer surgery
Source: Sci Rep. 2022 Jul 27;12:12871. doi: 10.1038/s41598-022-16975-w (PMC9329308; doi:10.1038/s41598-022-16975-w)
Supplement: Supplementary file 1 — Supplementary Table 1. [file 41598_2022_16975_MOESM1_ESM.docx]

|  | 405 nm | 415 nm | 434 nm | 465 nm | 494 nm | 520 nm | 542 nm | 572 nm | 605 nm |
| --- | --- | --- | --- | --- | --- | --- | --- | --- | --- |
| p-value | 0.53 | 0.37 | 0.83 | 0.53 | 0.47 | 0.47 | 0.52 | 0.44 | 0.48 |

**Supplementary Table 1: p-value of PNI- nerve compared to PNI+ nerve**
